# New Heterofunctional Supports Based on Glutaraldehyde-Activation: A Tool for Enzyme Immobilization at Neutral pH

**DOI:** 10.3390/molecules22071088

**Published:** 2017-06-29

**Authors:** Ricardo Rodrigues de Melo, Robson Carlos Alnoch, Adriana Ferreira Lopes Vilela, Emanuel Maltempi de Souza, Nadia Krieger, Roberto Ruller, Hélia Harumi Sato, Cesar Mateo

**Affiliations:** 1 Departamento de Biocatálisis, Instituto de Catálisis y Petroleoquímica (CSIC), Marie Curie 2. Cantoblanco, Campus UAM, 28049 Madrid, Spain; ricardorodriguesmelo@gmail.com (R.R.d.M.); robsonalnoch@hotmail.com (R.C.A.); dricea@hotmail.com (A.F.L.V.); 2 Laboratório Nacional de Ciência e Tecnologia do Bioetanol (CTBE), Centro Nacional de Pesquisa em Energia e Materiais (CNPEM), Cx. P. 6192, 13083-970 Campinas, São Paulo, Brazil; roberto.ruller@bioetanol.org.br; 3 Departamento de Ciência de Alimentos, Faculdade de Engenharia de Alimentos (FEA), Universidade Estadual de Campinas (UNICAMP), 13083-862 Campinas, São Paulo, Brazil; heliah@fea.unicamp.br; 4 Departamento de Bioquímica e Biologia Molecular, Universidade Federal do Paraná, Cx. P. 19081 Centro Politécnico, 81531-980 Curitiba, Paraná, Brazil; souzaem@ufpr.br; 5 Departamento de Química, Faculdade de Filosofia, Ciências e Letras de Ribeirão Preto, Universidade de São Paulo, 14040-901 Ribeirão Preto, São Paulo, Brazil; 6 Departamento de Química, Universidade Federal do Paraná, Cx. P. 19081 Centro Politécnico, 81531-980 Curitiba, Paraná, Brazil; nkrieger@ufpr.br

**Keywords:** enzyme immobilization, heterofunctional supports, glutaraldehyde, thermal stability, *Candida rugosa* lipase, metagenomic lipase, β-glucosidase, β-galactosidase

## Abstract

Immobilization is an exciting alternative to improve the stability of enzymatic processes. However, part of the applied covalent strategies for immobilization uses specific conditions, generally alkaline pH, where some enzymes are not stable. Here, a new generation of heterofunctional supports with application at neutral pH conditions was proposed. New supports were developed with different bifunctional groups (i.e., hydrophobic or carboxylic/metal) capable of adsorbing biocatalysts at different regions (hydrophobic or histidine richest place), together with a glutaraldehyde group that promotes an irreversible immobilization at neutral conditions. To verify these supports, a multi-protein model system (*E. coli* extract) and four enzymes (*Candida rugosa* lipase, metagenomic lipase, β-galactosidase and β-glucosidase) were used. The immobilization mechanism was tested and indicated that moderate ionic strength should be applied to avoid possible unspecific adsorption. The use of different supports allowed the immobilization of most of the proteins contained in a crude protein extract. In addition, different supports yielded catalysts of the tested enzymes with different catalytic properties. At neutral pH, the new supports were able to adsorb and covalently immobilize the four enzymes tested with different recovered activity values. Notably, the use of these supports proved to be an efficient alternative tool for enzyme immobilization at neutral pH.

## 1. Introduction

A simple, cheap and efficient immobilization strategy represents one of the main bottlenecks in the implementation of many industrial-scale enzymatic processes. Immobilization technology, as a very powerful tool, has been intensively applied to prepare various high-performance and economically-feasible biocatalysts improving activity, stability and selectivity [1,2,3,4]. By careful adjusting of the immobilization strategies, heterogeneous biocatalysts can work in broader pH and temperature ranges, and show greater thermal stability than their native free form [5,6].

Currently, a variety of methodologies for the immobilization of enzymes have been described. Among them, covalent immobilization on solid supports has been shown to be able to stabilize a variety of enzymes. Thus, supports activated with different functional groups (epoxy, aldehyde, glutaraldehyde, and others) have been developed and applied. However, the use of these supports only permit the random immobilization or the immobilization through only one region (e.g., richest in lysine region for aldehyde supports). This makes it so that only one kind of catalyst can be obtained using a functional covalent group; the product of the rigidification through one region or the average of the properties of the different enzyme molecules by random immobilization [7,8,9,10]. Because of these properties, several studies have shown the efficiency of immobilizing different enzymes in heterofunctionalized supports using various adsorbing groups (octyl, primary or secondary amino groups, ionic exchangers, metallic chelates and others) and reactive moieties (glyoxyl, epoxy, aldehyde, divinyl sulfone, amino, or glutamic groups), which are capable of adsorbing the protein physically and subsequently establishing covalent or ionic linkages with nucleophilic groups on the adsorbed protein [6,11,12,13,14,15,16]. One of the main advantages of use this type of supports is that they allows the orientation of the enzyme immobilization in different zones in its surface, being an alternative to the monofunctional supports. Heterofunctional supports are currently being studied as a useful tool in the immobilization of lipases (lipases from *Pseudomonas stutzeri*, *Rhizomucor miehei*, *Thermomyces lanuginosus*, and *Candida antarctica*), for instance, immobilizing silica or agarose supports containing distinct hydrophobic adsorbing groups (different alkyl groups) and reactive moieties [12,13,15,16,17]. In addition to the lipases, β-galactosidases (e.g., *Aspergillus oryzae* and *Thermus* sp.) and other enzymes have been immobilized on heterofunctional supports, obtaining good results with respect to their use [18,19,20,21]. For most strategies involving the application of heterofunctional supports, the immobilization process needs to be carried out in at least two steps: first the enzyme is adsorbed into the different adsorbent groups under mild conditions (i.e., low ionic strength and neutral pH), followed by elevation to alkaline conditions (around pH 10) for the occurrence of multiple interactions between nucleophilic groups on the enzyme (typically amino, hydroxyl, and/or thiol moieties) and reactive groups on the surface of the support. In some cases an additional step is carried out involving the reduction process necessary to establish multiple covalent linkages between the support and the enzyme [9]. However, the reduction step can cause some deleterious effects on the protein structure, cleaving disulfide and peptide linkages or reducing some groups essential for catalysis [22,23].

Although these immobilization methods are very useful, their uses have several limitations. One disadvantage of its use is the need to apply alkaline pH conditions (around pH 10) to enhance the reactivity of the nucleophilic residues found on the protein surface. Thus enzymes that are intrinsically unstable at alkaline pH values (e.g., the lipase from *Candida rugosa*) can be inactivated by the immobilization process [9,24,25]. An alternative process is the use of the reagent glutaraldehyde. The chemical covalent attachment process using glutaraldehyde is particularly attractive, since it provides a carefully regulated connection with specific groups found on the proteins under mild pH conditions [26]. Furthermore, the chemical reactivity shown by glutaraldehyde means that the reduction process with sodium borohydride is not strictly necessary [9]. 

Standard immobilization techniques using glutaraldehyde as the coupling agent are quite simple, efficient and probably the most used technique to carry out enzyme immobilization. Glutaraldehyde can react with different portions of the enzyme, mainly involving the primary amino groups of proteins, although it may eventually react with other groups (thiols, phenols and imidazoles) [9,26]. Supports functionalized with glutaraldehyde groups are described as being readily constructed from different supports containing primary amino groups (i.e., ethylenediamine-activated supports). However, the chemistry of glutaraldehyde in aqueous solution as well as the structures related to protein crosslinking or to enzyme immobilization are not yet fully understood. Some structures have been proposed for aqueous solutions of glutaraldehyde, which reported that in the pH from 3.0 to 8.0 and under dilute conditions, the glutaraldehyde reagent may exist as monomers (free aldehyde form or pre-dominantly cyclic hemiacetal), whereas in concentrations above 25% or under acidic conditions, oligomeric hemiacetals are formed. The equilibrium between the linear and cyclic monomeric forms is described as being linearly oriented as the temperature increases. Under basic conditions, oligomeric aldehydes may be formed by way of intermolecular aldol condensations [9,26,27].

Apart from the structural complexity presented by glutaraldehyde, its configuration already characterizes it as having distinct heterofunctionalities. Depending on the conditions applied during the process of immobilization with glutaraldehyde-activated supports, the proteins can be immobilized by three different mechanisms. On using very high ionic strength, the protein may first be immobilized by way of hydrophobic adsorption before forming the covalent bonds, whereas when using low ionic strength, primary immobilization will be by way of anion exchange. If the ionic strength applied were moderate, protein immobilization is described as being mainly due to covalent bonds [9,26,28,29,30].

**Scheme 1:**
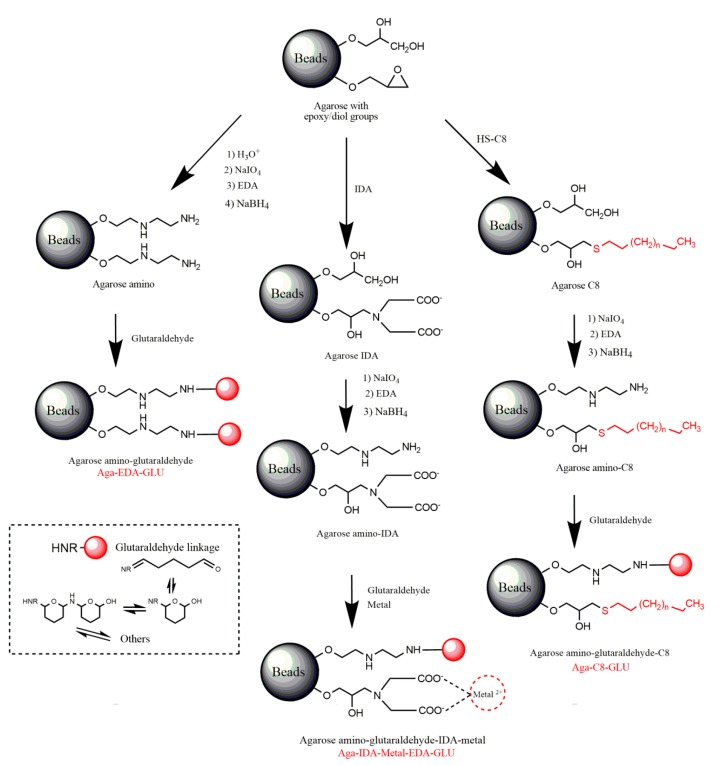
Schematic representation of the chemical modification in the surface of the agarose. The structures of the supports were idealized based on previous reviews concerning glutaraldehyde chemistry [26,27]. IDA (iminodiacetic acid); EDA (ethylenediamine); NaBH_4_ (sodium borohydride); NaIO_4_ (sodium periodate); GLU (glutaraldehyde); and HS-C8 (1-octanothiol).

In this work, a new generation of heterofunctional supports was proposed using different groups (hydrophobic or metal-chelate groups) capable of adsorbing proteins via different regions, followed by activation with the glutaraldehyde group, which can react covalently with the adsorbed proteins under neutral pH conditions (Scheme 1). Therefore, this work proposed the use of glutaraldehyde for the activation and formation of covalent linkages as a viable alternative to the alkaline mechanics currently applied. After the construction, a study was carried out with a multi-protein model system (*E. coli* BL21 crude extract) to define the conditions under which a pure immobilization mechanism occurred by way of adsorbed groups, and to define the conditions under which a connection can occur via only covalent bonds using the reagent glutaraldehyde. Structural analyses of the supports were carried out using Fourier Transformed Infrared (FTIR) spectroscopy with the aim of demonstrating the connection between the amine groups present in the support and the glutaraldehyde groups. Moreover studies using four different enzymes (*Candida rugosa* lipase—CRL, metagenomic lipase—LipC12, β-galactosidase—*Kl*Bgal and β-glucosidase—*Ea*BglA) were evaluated to verify the ability of the new supports to adsorb and form covalent linkages under neutral pH conditions. The properties of the immobilized enzymes, such as their activity and thermal stability, were also studied.

## 2. Results and Discussion

### 2.1. Construction and Analysis of New Heterofunctional Supports 

Agarose-based beads were chosen as a base matrix for the construction of different heterofunctional supports. Agarose is a strongly hydrophilic, lyophilic and inert colloid that can reversibly form stable and firm gels. Its suitability as an excellent support is confirmed by its high area rich in hydroxyl groups [31,32]. Briefly, agarose was activated with epichlorohydrin, a bifunctional reagent used to obtain epoxy groups [33]. The reaction was performed in alkaline conditions where most of the primary hydroxyl groups of the support were deprotonated. The primary reaction formed epoxy groups but as the reaction occurred in an alkaline environment, some of the epoxy groups created were hydrolyzed and yielded diol groups (Scheme 1). The total amount of activated primary hydroxyl groups obtained was around 65 ± 0.3 μmoL g^−1^, with epoxy groups accounting for 23 ± 0.4 μmoL g^−1^ and diol groups accounting for 42 ± 0.4 μmoL g^−1^. By obtaining a basic structure containing epoxy/diol groups, the new heterofunctional supports were prepared using the following strategy: (i) the epoxy groups formed were functionalized with different bifunctional reagents (i.e., hydrophobic or carboxylic/metal groups); and (ii) the diol groups were firstly oxidized with sodium periodate (NaIO_4_), and then activated with amino groups using ethylenediamine (EDA) (Scheme 1). The amino groups were applied to future activation with glutaraldehyde molecules. The new supports were constructed using very stable bifunctional groups and inert amino groups that allow storage for long periods prior to activation with the glutaraldehyde molecules.

**Figure 1:**
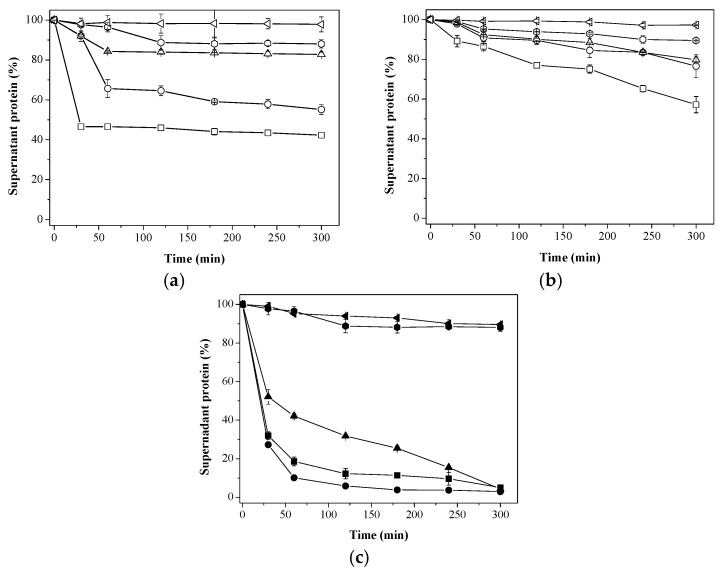
Immobilization of proteins present in the crude extract of *E. coli* BL21. (**a**) Immobilization on Aga-EDA support (Control); (**b**) Immobilization on Aga-EDA-GLU-Control support after the reduction process (Control). 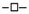 25 mM; 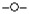 50 mM; 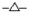 100 mM; 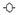 200 mM; and 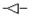 500 mM sodium phosphate buffer at pH 7; (**c**) Immobilization on different supports using 200 mM sodium phosphate buffer at pH 7. 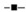 Aga-C8-GLU; 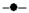 Aga-IDA-Ni^2+^-EDA-GLU; 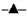 Aga-EDA-GLU; 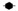 Aga-EDA (Control); and 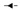 Aga-EDA-GLU-Control (Control). The results are expressed as the average of triplicate assays ± the standard error of the mean.

The new heterofunctional supports are expected to perform an initial protein adsorption only by the inserted heterofunctional groups (i.e., hydrophobic groups or metal chelate linked to the carboxylic groups), and only later to establish a covalent interaction with the glutaraldehyde group. However, reactive groups used in their construction, such as amino (from ethylenediamine, EDA), may interfere in a pure immobilization process. A conjugation chemistry that introduces a charged functional group into the support can cause nonspecific binding by promoting ion-exchange effects. However, in some cases, as in the traditional glutaraldehyde activated supports, a pure immobilization process is expected. Therefore, a linkage that alters the flow and binding characteristics of the support, e.g., EDA, is not of interest for the work. Thus, a possible unspecific adsorption via ion-exchange by the amino groups was studied. A multi-protein model system (*E. coli* BL21 crude extract, 5 mg g^−1^ of support) was offered to a pure amino support (activated only with ethylenediamine/Aga-EDA), and then the adsorption process at different ionic strengths was analyzed (Figure 1a). The *E. coli* extract was applied because it contains proteins with different properties and characteristics, therefore providing different affinities for the adsorption [30]. At pH 7 and low ionic strength (25 mM sodium phosphate buffer), 50% of proteins were quickly adsorbed in 30 min (Figure 1a). An increase in the ionic strength promoted a decrease in the adsorption by amino groups until a negligible adsorption of proteins was found in 500 mM sodium phosphate buffer (even at 200 mM sodium phosphate buffer, the adsorption was almost negligible during the first hour). The results show that the new supports could not be used under low ionic strength due to protein interaction being conducted via a double mechanism, which would be promoted by amino groups (from ethylenediamine), and by the heterofunctional groups inserted in the new supports. 

Similarly, a possible adsorption by the glutaraldehyde structure was studied. Depending on the conditions applied, the immobilization process with glutaraldehyde groups can occur by a double step: in a first step, there is the adsorption of proteins on the support and then, the covalent immobilization [26,30]. Thus, a study using a support activated only by glutaraldehyde groups but reduced with sodium borohydride (Aga-EDA-GLU-Control) was done. The reduction step with sodium borohydride was used to transform the Schiff’s bases (–N=C– double bond) into stable primary amino bonds [34]. A negligible adsorption was observed using either 200 mM or 500 mM sodium phosphate buffer (Figure 1b). On the other hand, when low ionic strengths were applied, a significant protein adsorption was found (21% in 100 mM). Therefore, the results indicate that the process of the immobilization on the new heterofunctional supports has to be carried out at a moderate ionic strength (200–500 mM) to prevent unspecific interaction, such as ion-exchange. Furthermore, it has been described that protein immobilization using glutaraldehyde groups with moderate ionic strength occur by direct covalent attachment [9,26,35], which is of interest in the present study.

Considering these data, the *E. coli* BL21 crude extract was offered to new supports using moderate ionic strength (200 mM sodium phosphate buffer at pH 7) to prevent unspecific absorptions, and to promote a direct covalent and irreversible immobilization by glutaraldehyde groups. Using the new supports (Aga-C8-GLU and Aga-IDA-Metal-EDA-GLU), more than 95% of all proteins from *E. coli* BL21 crude extract were immobilized after 5 h (Figure 1c). To compare the immobilization efficiency of the new supports, the crude extract was offered to the traditional support activated using glutaraldehyde (Aga-EDA-GLU), and a similar result was obtained after 5 h (Figure 1c). The controls (Aga-EDA and Aga-EDA-GLU-Control) were repeated and no protein adsorption was found (Figure 1c). The findings suggest that the proteins can be immobilized on new glutaraldehyde-activated supports at moderate ionic strength, where unspecific interactions are not found.

**Figure 2:**
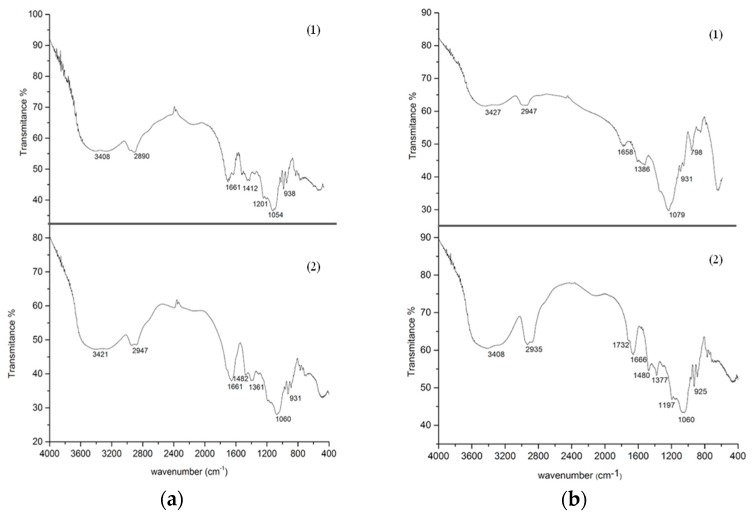
Fourier Transformed Infrared (FTIR) spectra for agarose supports: (**a**) Aga-IDA-EDA support (1) before modification, and (2) after chemical modifications with the agent glutaraldehyde; and (**b**) Aga-C8-EDA support, (1) before modification, and (2) after chemical modifications with the agent glutaraldehyde. Aga (agarose beads); IDA (iminodiacetic acid); EDA (ethylenediamine); and C8 (1-octanothiol).

FTIR spectra analysis was used to characterize the activation with the glutaraldehyde group, reflecting the effectiveness of the developed procedure in producing different glutaraldehyde-activated supports. The FTIR spectra were taken from untreated and treated with the glutaraldehyde group, using EDA-functionalized supports to determine any changes in chemical bonding states (Figure 2). The new band around 1660 cm^−1^ (as shown in Figure 2a(2) and Figure 2b(2)) was ascribed to the presence of imine (–N=C–) in the support as the result of the reaction between glutaraldehyde and the amino groups of the EDA found on the supports. Previous studies indicate that the reaction of the agent glutaraldehyde with primary amine groups in the treated support is well accepted [27,36].

### 2.2. Immobilization of Different Biocatalysts on the New Heterofunctional Supports

The new supports were used for the covalent immobilization of different enzymes in mild conditions (neutral pH). For the immobilization assays on these supports, four enzymes of biotechnological interest were used, namely: *Candida rugosa* lipase (CRL), metagenomic lipase (LipC12), β-galactosidase (*Kl*Bgal) and β-glucosidase (*Ea*BglA).

To prevent non-specific adsorptions, the immobilization tests were carried out using a moderate ionic strength (200 mM at pH 7). In all cases, the non-specific adsorption was negligible. Table 1 shows the tests with the different glutaraldehyde-activated supports using the lipase from *Candida rugosa* (CRL). Lipase CRL is an enzyme used in several biocatalytic processes; however, it shows great structural instability especially at alkaline pH [25,37]. At pH 7, immobilization tests with lipase CRL on the new glutaraldehyde-activated supports were done, and the data showed that the final recovered activity was strongly dependent on the support used. The recovered activity (R) values were from 210% (new hydrophobic group-glutaraldehyde support, Aga-C8-GLU) to 54% (new metal group-glutaraldehyde support, Aga-IDA-Metal-EDA-GLU). Traditional glutaraldehyde support (Aga-EDA-GLU) showed almost unaltered recovered activity (R = 95%) compared to the initial activity of the free enzyme. For further studies, lipase CRL was also immobilized on a commercial support activated only with hydrophobic groups (Octyl-Sepharose). The commercial hydrophobic support showed a recovered activity of R = 225%, similar to that found by the new hydrophobic group-glutaraldehyde support (Aga-C8-GLU, Table 1). Both hydrophobic supports have shown a hyperactivation described as being related to the interfacial activation of the enzyme, whereby the catalyst may be adsorbed in its open form, compared to the soluble form in the standard conformational equilibrium [38]. Thus, the presence of the agent glutaraldehyde on Aga-C8-GLU did not significantly alter lipase adsorption by the hydrophobic group. The results for lipase CRL immobilized on the Aga-C8-GLU were in general agreement with a previous study with octyl activated with divinyl sulfone (OCDVS, more than 3-fold recovered activity) [14]. 

**Figure 3:**
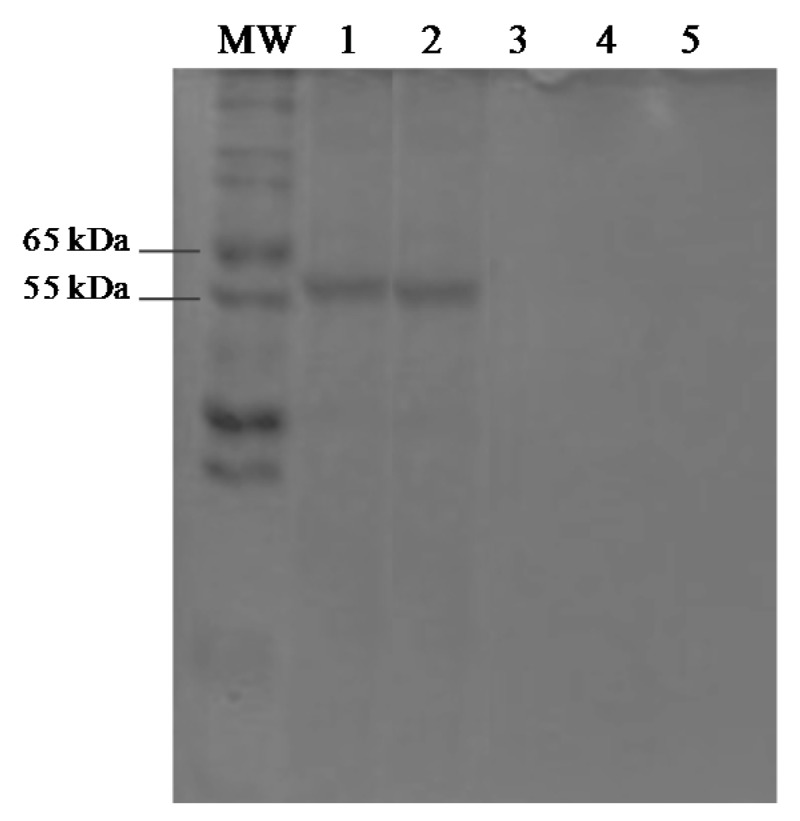
SDS-polyacrylamide gel electrophoresis (SDS-PAGE) analysis of CRL desorption assays from different supports. The lanes were loaded as follows: lane MW, protein molecular weight markers; lane 1, free CRL lipase; **lane 2**, eluted fraction from the commercial Octyl-Sepharose; **lane 3**, eluted fraction from Aga-EDA-GLU at pH 7; **lane 4**, eluted fraction from Aga-C8-GLU at pH 7; **lane 5**, eluted fraction from Aga-IDA-Ni^2+^-EDA-GLU at pH 7. The proteins were stained with Coomassie Brilliant Blue R-250.

To analyze if CRL was covalently attached to the new supports and not only by adsorption, the different CRL preparations were boiled in Laemmli’s disruption buffer (which contains mercaptoethanol and SDS) and analyzed by SDS-polyacrylamide gel electrophoresis (SDS-PAGE, Figure 3). CRL was used as a model because it is a monomeric enzyme. The glutaraldehyde is known to establish covalent bonds at pH 7 [9,39]. According to SDS-PAGE results, the new glutaraldehyde-activated supports did not allow the release of the immobilized CRL, proving that the new supports can promote the adsorption and subsequent covalent immobilization by the glutaraldehyde group at pH 7. CRL immobilized on Octyl-Sepharose produced a band in SDS-PAGE (Figure 3), which indicates only the adsorption to the hydrophobic groups.

Further experiments of lipase CRL desorption from the different supports were done by the addition of Triton ×100, NaCl and imidazol (Table 2). The tests were used to prove that lipase CRL was not hydrophobic or ion-bound to the new supports. In the analysis, the activities of supernatants and suspensions were measured to verify the desorption of the immobilized CRL. The use of the non-ionic detergent Triton ×100 produced a significant desorption of the immobilized CRL on Octyl-Sepharose (81% initial activity), while a negligible activity was obtained in the supernatant for the new hydrophobic group-glutaraldehyde support/Aga-C8-GLU (Table 2). Higher concentrations of Triton ×100 were not able to remove CRL from the new glutaraldehyde-activated support (data not shown). Analysis using NaCl and imidazol showed a negligible activity found in the supernatant for the immobilized CRL on Aga-EDA-GLU and Aga-IDA-Metal-EDA-GLU supports. These results confirm the covalent immobilization of lipase CRL by the glutaraldehyde group at pH 7.

Another monomeric lipase obtained from metagenomics (lipase LipC12, [40]) was also studied (Table 1). All the assayed supports allowed the complete immobilization of lipase LipC12. The recovered activities were different depending on the support used in the immobilization (Table 1). Upon immobilization on all supports, a high-recovered activity (R = 269%) was obtained with the new hydrophobic group-glutaraldehyde support (Aga-C8-GLU) showing a hyperactivation of lipase LipC12 (Table 1). Moreover, LipC12 immobilized on Aga-IDA-Metal-EDA-GLU and Aga-EDA-GLU (traditional support) showed recovered activity of 100% and 42%, respectively. The traditional glutaraldehyde-activated support used as control system showed a low recovered activity of LipC12 in relation to the new supports. A similar study of covalent attachment by the glutaraldehyde group was accomplished, and no desorption was found for LipC12 immobilized on the new supports (data not shown).

Tests analyzing the possibility of using the new glutaraldehyde-activated supports on the immobilization of two complex multimeric enzymes were studied. β-galactosidase (*Kl*Bgal) and β-glucosidase (*Ea*BglA) are described as tetrameric enzymes [41,42]. The structural complexity of these catalysts represents an especially complex problem for immobilization because small disturbances in their quaternary-structures may alter the precise distribution of the native state ensemble [39,43], with dissociation of subunits producing enzymatic inactivation. Therefore, these enzymes are fragile even in mild conditions. Table 3 shows the results for *Kl*Bgal and *Ea*BglA with a final recovered activity ranging from 81 to 70% for the β-galactosidase and from 64 to 0% for the β-glucosidase. *Kl*Bgal immobilized on the new supports showed recovered activity values of 81% (Aga-C8-GLU) and 78% (Aga-IDA-Co^2+^-EDA-GLU). The traditional GLU supports (Aga-EDA-GLU) had an R 70%. The traditional glutaraldehyde-activated support (control system) showed a low recovered activity of *Kl*Bgal in relation to the new supports. A similar result has been reported for the covalent immobilization of *Kluyveromyces lactis* β-galactosidase in glyoxyl-Sepharose support [20], retaining approximately 82% of its initial activity after immobilization. In the case of *Ea*BglA, Aga-IDA-Co^2+^-EDA-GLU showed the highest R (64%), following the traditional Aga-EDA-GLU with R 50%. The new hydrophobic-glutaraldehyde support yielded an inactive derivative after the immobilization process. Zanphorlin et al. [42] describe that the selective pressure at low temperatures favored mutations that redesigned the *Ea*BglA protein surface, reducing the number of salt bridges, and exposing more the hydrophobic regions to the solvent. Its can thus be concluded that one reason for the inactivation of *Ea*BglA when immobilized in the hydrophobic support may be due to the interaction between a high number of hydrophobic groups in the protein surface and the hydrophobic group of the support. As in the other cases, no activity was detected after desorption of the different preparations after incubation in Triton ×100, NaCl and imidazol.

### 2.3. Thermal Stability 

**Figure 4:**
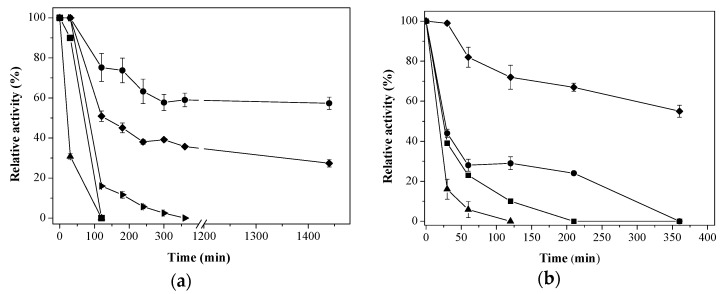
Thermal stability. (**a**) Lipase CRL at pH 7, 50 °C. (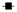) Aga-IDA-Metal-EDA-GLU; (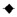) Aga-C8-GLU; (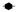) Aga-EDA-GLU; (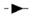) Octyl-Sepharose; and (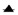) free enzyme; (**b**) Lipase LipC12 at pH 7, 70 °C. (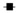) Aga-IDA-Metal-EDA-GLU; (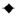) Aga-C8-GLU; (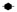) Aga-EDA-GLU; and (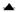) free enzyme. The results are expressed as the average of triplicate assays ± the standard error of the mean.

Enzymatic stabilization has great importance due to the increasing number of applications of enzymes in almost all areas and it is one of the main aims when the immobilization of enzymes is performed [6]. Therefore, the thermal stabilities of different immobilized preparations and free forms were studied in 100 mM phosphate buffers, pH 7 using distinct temperatures. Figure 4a shows the tests using lipase CRL preparations. The stability of the adsorbed preparation (Octyl-Sepharose) was considerably similar to the free form with around 3.6-fold stabilization. The maximal stability was obtained with the traditional glutaraldehyde support (Aga-EDA-GLU) with a half-life of around 1560 min after incubation at 50 °C (Table 4). Considering that the half-life of the free CRL was 21 min, the stabilization factor obtained was around 74.3-fold. CRL immobilized on Aga-C8-GLU and Aga-IDA-Ni^2+^-EDA-GLU was 5.7 and 2.8-fold more stable than the free form.

The thermal stability of lipase LipC12 preparations was assessed by incubation at 70 °C (Figure 4b, Table 4). At this temperature, the native free LipC12 had a half-life time of 13 min. The most stable preparation was found using the new hydrophobic group-glutaraldehyde support (Aga-C8-GLU) with a stabilization factor around 32.3-fold compared to the free form. The stability of LipC12 immobilized on the traditional amino (Ag-EDA-GLU) and new metal group-glutaraldehyde support (Aga-IDA-Ni^2+^-EDA-GLU) was, respectively, 5.8 and 1.8-fold higher than the free LipC12. The high improvement of lipase LipC12 after immobilization (Aga-C8-GLU/LipC12) is important because it permits the transformation of a mesophilic enzyme into an enzyme with properties that are similar to those of thermophilic organisms, such as *Bacillus thermocatenolatus* lipase (BTL) and *Thermus thermophilus* lipase (TTL) [44,45].

**Figure 5:**
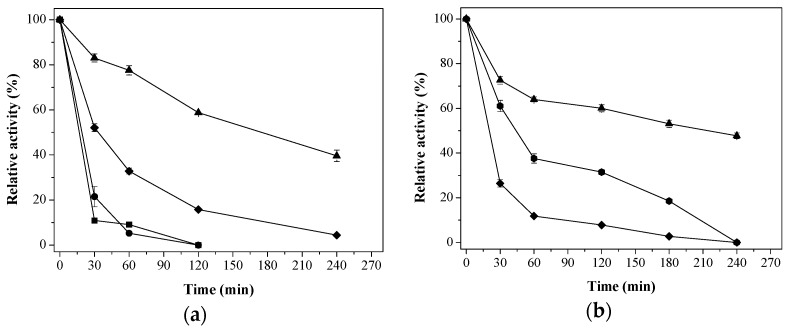
Thermal stability. (**a**) β-galactosidase (*Kl*gal) at pH 7, 40 °C. (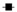) Aga-C8-GLU; (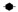) Ag-EDA-GLU; (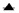) Aga-IDA-Metal-EDA-GLU, and (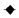) free enzyme; (**b**) β-glucosidase (*Ea*glA) at pH 7, 40 °C. (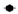) Ag-EDA-GLU; (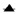) Aga-IDA-Metal-EDA-GLU, and (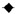) free enzyme. The results are expressed as the average of triplicate assays ± the standard error of the mean.

Figure 5 shows the thermal stability of the immobilized *Kl*Bgal and *Ea*BglA at 40 °C. For *Kl*Bgal preparations, only Aga-IDA-Co^2+^-EDA-GLU promoted a significant stabilization (4.8-fold factor) compared to the free form (half-life of 33 min at 40 °C, Figure 5a and Table 4). All other preparations showed a lower stabilization than the free form (Aga-C8-GLU 0.52 and Aga-EDA-GLU 0.36-fold factor, Table 4). For studies with the immobilized *Ea*BglA, it was observed that the Aga-IDA-Co^2+^-EDA-GLU (half-life time of 230 min) showed the best result, which was more stable (5.9-fold factor) than the traditional GLU preparation (Aga-EDA-GLU), and 10.9-times more stable than free enzyme (half-life time of 21 min at 40 °C, Figure 5b and Table 4). The Aga-EDA-GLU preparation also presents a half-life of 39 min (1.8-fold factor more stable than free form). In all cases, the stability of different preparations was strongly dependent on the support used in the immobilization process.

## 3. Materials and Methods 

### 3.1. Materials

Agarose 4 BCL was purchased from Agarose Bead Technologies (Madrid, Spain). Epichlorohydrin, iminodiacetic acid, triethylamine, sodium borohydride, sodium periodate, 1-octanothiol, bovine serum albumin, *O*-nitro-phenyl-β-d-galactopyranoside (*O*-NPG), *p*-nitrophenyl proprionate (*p*-NPP), 4-nitrophenyl-β-d-glucopyranoside (*p*-NPG), and high molecular weight protein (Sigma Marker^TM^, St. Louis, MO, USA) were purchased from Sigma (Sigma-Aldrich^®^, St. Louis, MO, USA). Glutaraldehyde solution (25%, *v*/*v*) and ethylenediamine were purchased from Alfa Aesar (Thermo Fisher Scientific^®^, Waltham, MA, USA). Octyl-Sepharose CL-4B was purchased from GE Healthcare Bio-Sciences (Uppsala, Sweden). The β-galactosidase from *Kluyveromyces lactis* (Lactozym pure 6500 L) (*Kl*Bgal) was kindly supplied by Novozymes A/S (Copenhagen, Denmark). The lipase from *Candida rugosa* (CRL) was purchased from Sigma (Sigma-Aldrich^®^, St. Louis, MO, USA). Overexpression and purification of lipase (LipC12) and β-glucosidase (*Ea*BglA) were performed as previously described by Glogauer et al. [40] and Crespim et al. [47], respectively. All other chemicals used were of analytical grade.

### 3.2. Standard Determination of Enzymatic Activities

β-Glucosidase *assays.* The activity was determined using 4-nitrophenyl β-d-glucopyranoside (*p*-NPG) as substrate. The free or immobilized enzyme was added to a mixture solution (5 mM *p*-NPG in 50 mM sodium phosphate buffer at pH 7) and the increase in absorbance was monitored at 410 nm (pH 7.0, *ε*_410 nm_ = 7320 M^−10^ cm^−1^) [47]. One unit (U) of enzyme was defined as the amount of enzyme required to release1 μmoL of *p*-nitrophenol per min and the specific activity was defined as the number of units per mg of protein.

*Lipase assays.* The activities were determined using *p*-nitrophenyl propionate (*p*-NPP) as substrate. The free or immobilized enzymes were added to a mixture solution (0.4 mM *p*-NPP in 50 mM sodium phosphate buffer at pH 7) and the increase in absorbance was monitored at 348 nm (pH 7.0, *ε*_348 nm_ = 5150 M^−1^ cm^−1^) [29]. One unit (U) of enzyme was defined as the amount of enzyme required to release 1 μmoL of *p*-nitrophenol per min and the specific activity was defined as the number of units per mg of protein.

β-Galactosidase *assays*. The activity was determined using *O*-nitro-phenyl-β-d-galactopyranoside (*O*-NPG) as substrate. The free or immobilized enzyme was added to a mixture solution (5 mM *O*-NPG in 50 mM potassium phosphate buffer at pH 7 with the addition of 2 mM MgCl_2_) and the increase in absorbance was monitored at 410 nm (pH 7.0, *ε*_410 nm_ = 3500 M^−1^ cm^−1^) [48]. One unit (U) of enzyme was defined as the amount of enzyme required to release 1 μmoL of *O*-NP per min and the specific activity was defined as the number of units per mg of protein.

### 3.3. Supports for Immobilization

#### 3.3.1. Activation of Agarose-Based Beads

Epoxy-activated agarose was prepared by the reaction of the hydroxyl groups on the agarose beads with epichlorohydrin, as described by Mateo et al. [24]. Under gentle agitation and in an ice bath, 50 g of agarose 4 BCL, previously washed with distilled water and vacuum dried, was mixed with 220 mL of distilled water, 16.4 g of NaOH, 1.0 g of sodium borohydride (NaBH_4_), 80 mL of acetone, and 55 mL of epichlorohydrin. The suspension was incubated for 16 h at 25 °C, and then washed with the excess of distilled water, vacuum dried, and stored at 4 °C.

The number of epoxy/ligand groups was calculated from the difference in periodate consumption between the hydrolyzed support and the initial epoxy support. Periodate consumption was quantified using potassium iodide, as previously described [49].

#### 3.3.2. Modification of the Epoxy-Activated Agarose with Different Reactive Groups

##### Cationic Supports

Epoxy-activated agarose (10 g) was modified using 1 M triethylamine solution (acetone/water, 50:50 *v*/*v*) for 18 h at 25 °C [24]. After this, the support was oxidized with 100 mL of 100 mM sodium periodate (NaIO_4_) for 2 h at 25 °C, washed with distilled water, vacuum dried and stored at 4 °C.

##### Support Activated with the Glutaraldehyde Group

The support activated using the glutaraldehyde group was prepared as previously described by Betancor et al. [35]. Briefly, the epoxy-activated agarose (10 g) was hydrolyzed with 100 mL of 1 M H_2_SO_4_ for 2 h at 25 °C. Afterwards, the support was washed with distilled water and oxidized using 100 mL of 100 mM NaIO_4_. Then, the support was treated with 100 mL of 2 M ethylenediamine (EDA) at pH 10, and kept under gentle stirring at 25 °C. After 2 h, sodium borohydride (10 mg mL^−1^) was added and stirred for further 2 h at 25 °C. The particles were successively washed with distilled water and 11 mL of glutaraldehyde solution (25%, *v*/*v*) was added together with 17 mL of sodium phosphate buffer (200 mM at pH 7). The system was kept under gentle stirring for 18 h at 25 °C. Finally, the activated support was washed with distilled water and vacuum dried.

##### Anionic Supports Activated with the Glutaraldehyde Group and Metal Chelate

Agarose support (10 g) activated with the glutaraldehyde group and metal chelate was obtained by treatment of the epoxy-activated agarose with 100 mL of 500 mM iminodiacetic acid (IDA) at pH 11 for 18 h at 25 °C [24]. Then, the support was washed with distilled water and oxidized using 100 mL of sodium periodate (NaIO_4_) at a final concentration of 100 mM. After 2 h of gentle agitation at 25 °C, the oxidized support was washed with distilled water and treated with 100 mL of 2 M ethylenediamine (EDA) pH 10 for 2 h at 25 °C. Sodium borohydride (10 mg mL^−1^) was added, and stirred for a further 2 h at 25 °C. The activated agarose was successively washed with distilled water. Glutaraldehyde solution (25%, *v*/*v*) and sodium phosphate buffer (200 mM at pH 7) were added, and the system was kept under gentle stirring for 18 h at 25 °C. The agarose activated with glutaraldehyde was added to the metal chelate solutions (30 mg mL^−1^, CoCl_2_ or NiCl_2_ 6 H_2_O) at pH 7 for 30 min at 25 °C. The activated support was washed with distilled water and vacuum dried.

##### Hydrophobic Supports Activated with the Glutaraldehyde Group

Agarose activated with the hydrophobic group and glutaraldehyde was prepared by treatment of the epoxy-activated agarose with 100 mL of 100 mM 1-octanothiol in NaHCO_3_ (25 mM) at pH 10 for 24 h at 25 °C. Thereafter, the support was oxidized with 100 mL of NaIO_4_ (100 mM), washed, and filtered using a glass filter. The hydrophobic support was then treated with 100 mL of 2 M ethylenediamine (EDA), pH 10 for 2 h at 25 °C, following the addition of sodium borohydride (10 mg mL^−1^) for 2 h at 25 °C. After it was washed and dried, the glutaraldehyde solution (25%, *v*/*v*) and sodium phosphate buffer (200 mM at pH 7.0) were added. The activated support was washed with distilled water and vacuum dried.

### 3.4. Immobilization of Enzymes

For all immobilization experiments, β-glucosidase (*Ea*BglA) and lipases (CRL and LipC12) were solubilized in 200 mM sodium phosphate buffer at pH 7, and β-galactosidase (*Kl*Bgal) was solubilized in 200 mM potassium phosphate buffer at pH 7 with the addition of 2 mM MgCl_2_. Then, different immobilization supports were suspended in an enzyme solution (1 g support: 10 mL of enzyme solution in the immobilization buffer, 1 mg g^−1^ of support). The immobilization suspensions were gently stirred at 25 °C. Periodically, samples of supernatants were withdrawn, and their enzymatic activities were analyzed. The immobilization was considered complete when there was no activity in the supernatant. 

After immobilization, the enzymes were washed with phosphate buffers. The immobilization efficiency (*IE*, %) was calculated as Equation (1):

(1) $$IE=\frac{A_{i}-\;A_{f}}{A_{i}}\;\times \;100\%$$

where *Ai* is the amount activity (U) of the enzyme solution before immobilization and *A_f_* is the amount activity (U) remaining in the supernatant at the end of the immobilization procedure. 

The recovered activity (*R*, %) was calculated as Equation (2):

(2) $$R=\frac{A_{o}}{A_{T}}\times \;100\%$$

where *A_o_* is the as the ratio between the real specific activity (U g^−1^ of support) of immobilized preparation and *A_T_* is the theoretical specific activity of the immobilized preparation (U g^−1^ of support).

*Escherichia coli* BL21 crude extract was used as a multi-protein model system during the immobilization tests with the different supports. One gram of activated supports was suspended in 10 mL of *E. coli* BL21 crude cell extract (5 mg g^−1^ of support). The adsorption of proteins was monitored using the Bradford method.

### 3.5. Protein Assays and SDS-PAGE

Protein content was determined by the Bradford method [50], using a Coomassie Protein Assay Kit (Pierce Biotechnology, Rockford, IL, USA) with bovine serum albumin as the standard. Electrophoresis of protein samples was done with 12% (*w*/*v*) SDS-PAGE [51]. The gel was stained with Coomassie Brilliant Blue R-250 and distained with methanol/acetic-acid/water (5/1/4 *v*/*v*/*v*). A mixture of high molecular weight proteins (Sigma Marker™, Sigma-Aldrich^®^, St. Louis, MO, USA) was used as the molecular weight standard.

### 3.6. Fourier Transform Infrared (FTIR)

Identification of the chemical groups on the untreated and treated support was performed using the FTIR spectroscopy Vertex 70 Bruker. The spectra were obtained with a wave number range from 4000 to 400 cm^−1^ at a resolution of 4 cm^−1^ over 32 cumulative scans. FTIR spectra were not used as a quantitative relationship, but only as a qualitative reference.

### 3.7. Desorption Process Analysis

Samples of 1 g of immobilized enzymes were suspended in 3 mL of phosphate buffer (5 mM, at pH 7). Then, Triton ×100, NaCl and imidazol were added to a final concentration of 0.5% (*v*/*v*), 1.0 M and 0.5 M, respectively. The different preparations were gently stirred at 25 °C and, periodically, samples from supernatants were withdrawn and their enzymatic activities were analyzed.

### 3.8. Thermal Stability

Thermal stabilities were assessed by incubation in sodium phosphate buffer (100 mM, pH 7 at 40 °C) for β-glucosidase, potassium phosphate buffer containing 2 mM MgCl_2_ (100 mM, pH 7 at 40 °C) for β-galactosidase, and sodium phosphate buffer (100 mM, pH 7 at 50 °C or 70 °C) for lipases (CRL and LipC12). Aliquots were periodically withdrawn for the quantification of the residual enzymatic activity to estimate the half-life time according to Henley and Sadana [46]. The relative activities of free and immobilized forms without incubation were defined as control and attributed a relative activity of 100%. Inactivation parameters were determined from the best-fit model of the experimental data which was the one based on a two-stage series inactivation mechanism with residual activity. Half-lives (time in which the residual enzyme activity is half of its initial value) were used to compare the stability of the different preparations, being determined by the interpolation from the respective models described in [52].

## 4. Conclusions

The new generation of supports proposed in this study overcomes some of problems associated with the immobilization of enzymes intrinsically unstable at alkaline pH. The new supports were capable of immobilizing distinct enzymes with significant recovered activity values. Thus, this strategy allowed obtaining heterogeneous catalysts with different catalytic properties (i.e., activity and stability) under mild conditions (neutral pH). 

The strategy of this study can be applied to other supports activated in similar ways, such as cellulose, permitting the improvement of the materials or the use of supports with distinct morphologies or properties.

## Figures and Tables

**Table 1:** *Candida rugosa* lipase (CRL) and metagenomic lipase (LipC12) immobilization on glutaraldehyde-activated supports.

Enzyme	Support	Immobilization Efficiency ^a^ (%), IE	Recovered Activity after Immobilization at pH 7 ^b^ (%), R
	Aga-C8-GLU	100	210
**CRL**	Aga-IDA-Ni^2+^-EDA-GLU	95	54
	Aga-EDA-GLU	100	95
	Octyl-Sepharose	100	225
	Aga-C8-GLU	100	269
**LipC12**	Aga-IDA-Ni^2+^-EDA-GLU	100	100
	Aga-EDA-GLU	100	42

C8 (1-octanothiol); Ni^2+^ (nickel); EDA (ethylenediamine); IDA (iminodiacetic acid); Aga (agarose beads); and GLU (glutaraldehyde). ^a^ Calculated as the difference between the initial and final activities in the supernatant after 1 h of immobilization. ^b^ Recovered activity (%), measured as the ratio between the real activity (U g^−1^ of support) of immobilized enzymes (CRL or LipC12) and theoretical activity of the immobilized enzymes (CRL or LipC12) (U g^−1^ of support).

**Table 2:** Desorption assays of different CRL preparations.

Support	Immobilization Efficiency ^a^ (%), IE	Recovered Activity after Incubation ^b^ (%), R	Activity in the Supernatant after Incubation (%)
Aga-EDA-GLU *	100	97	0
Aga-C8-GLU **	100	90	0
Octyl-Sepharose **	100	12	81
Aga-IDA-Ni^2+^-EDA-GLU ***	100	98	0

C8 (1-octanothiol); Ni^2+^ (nickel); EDA (ethylenediamine); IDA (iminodiacetic acid); Aga (agarose beads); and GLU (glutaraldehyde). ^a^ Calculated as the difference between the initial and final activities in the supernatant after 1 h of immobilization. ^b^ Recovered activity (%), measured as the ratio between the real activity (U g^−1^ of support) of immobilized lipase CRL and theoretical activity of the immobilized lipase CRL (U g^−1^ of support). * Preparation incubated in 1 M NaCl; ** Preparation incubated in 0.5% of Triton ×100; *** Preparation incubated in 0.5 M imidazol.

**Table 3:** β-Galactosidase (*Kl*gal) and β-glucosidase (*Ea*glA) immobilization on glutaraldehyde-activated supports.

Enzyme	Support	Immobilization Efficiency ^a^ (%), IE	Recovered Activity after Immobilization at pH 7 ^b^ (%), R
	Aga-C8-GLU	100	81
*Kl*Bgal	Aga-IDA-Co^2+^-EDA-GLU	>94	78
	Ag-EDA-GLU	100	70
	Aga-C8-GLU	100	0
*Ea*BglA	Aga-IDA-Co^2+^-EDA-GLU	100	64
	Aga-EDA-GLU	100	50

C8 (1-octanothiol); Co^2+^ (cobalt); EDA (ethylenediamine); IDA (iminodiacetic acid); Aga (agarose beads); and GLU (glutaraldehyde). ^a^ Calculated as the difference between the initial and final activities in the supernatant after 1 h of immobilization. ^b^ Recovered activity (%), measured as the ratio between the real activity (U g^−1^ of support) of immobilized enzymes (*Kl*Bgal or *Ea*BglA) and theoretical activity of the immobilized enzymes (*Kl*Bgal or *Ea*BglA) (U g^−1^ of support).

**Table 4:** Half-life (in minutes) of different immobilized preparations.

Enzyme	Support	Half-Life (T_1/2_, Minutes)	Stability Factor
**CRL ^a^**	Free form	21	
	Aga-C8-GLU	120	5.7
	Aga-IDA-Ni^2+^-EDA-GLU	60	2.8
	Aga-EDA-GLU	1560	74.3
	Octyl-Sepharose	76	3.6
**LipC12 ^b^**	Free form	13	
	Aga-C8-GLU	420	32.3
	Aga-IDA-Ni^2+^-EDA-GLU	23	1.8
	Aga-EDA-GLU	76	5.8
***Kl*Bgal ^c^**	Free form	33	
	Aga-C8-GLU	17	0.52
	Aga-IDA-Co^2+^-EDA-GLU	160	4.8
	Aga-EDA-GLU	12	0.36
***Ea*BglA ^c^**	Free form	21	
	Aga-C8-GLU	-	-
	Aga-IDA-Co^2+^-EDA-GLU	230	10.9
	Aga-EDA-GLU	39	1.8

C8 (1-octanothiol); Ni^2+^ (nickel); Co^2+^ (cobalt); EDA (ethylenediamine); IDA (iminodiacetic acid); Aga (Agarose beads); and GLU (glutaraldehyde). ^a^ Thermal stability of lipase CRL preparations was checked by incubation at 50 °C. ^b^ Thermal stability of lipase LipC12 preparations was checked by incubation at 70 °C. ^c^ Thermal stability of *Kl*gal and *Ea*glA preparations was checked by incubation at 40 °C. Aliquots were periodically withdrawn for quantification of the residual enzymatic activity to estimate the half-life according to Henley and Sadana [46]. The stability factors were done in relation to the free form.
